# Surveillance of the impact of pneumococcal conjugate vaccines in developing countries

**DOI:** 10.1080/21645515.2015.1057671

**Published:** 2015-08-26

**Authors:** Gail L Rodgers, Keith P Klugman

**Affiliations:** Pneumonia Program; The Bill and Melinda Gates Foundation; Seattle, WA USA

## Abstract

Infection due to Streptococcus pneumoniae is a leading cause of morbidity and mortality in young children, especially in developing countries. With the support of Gavi, the Vaccine Alliance, the majority of these countries have introduced pneumococcal conjugate vaccines (PCV) into their national immunization programs and early data demonstrate a high degree of effectiveness, translating to enormous public health benefit through both direct and indirect (herd) effects. Future vaccination strategy may be focused on maintaining herd effects rather than individual protection. Evaluation of vaccine-type carriage, particularly in pneumonia cases, may be an easy, feasible way of measuring continued vaccine impact.

The developing world accounts for a disproportionate amount of the global pneumococcal disease burden and the vast majority of *Streptococcus pneumoniae* (SP) related deaths.^1^ Therefore, use of currently available pneumococcal conjugate vaccines (PCVs) in children, particularly in the developing world is critical.

In 2000, the first conjugate pneumococcal vaccine, containing 7 serotypes was licensed. Use of PCV7, mostly in the high income countries, resulted in dramatic reductions in all manifestations of pneumococcal disease and its mortality. In view of PCV7 effectiveness, in 2007 the WHO recommended incorporation of PCVs into the national immunization programs of all countries, especially in countries with high childhood pneumococcal disease (PD) burden.^2^ Expanded PCVs containing 10 and 13 serotypes were licensed a decade after PCV7 and importantly, these new PCVs expanded the coverage to include the most common disease-causing serotypes in the developing world. Recognizing the PD burden and associated mortality, Gavi, the Vaccine Alliance, with support from international donors, created the pneumococcal advance market commitment (AMC) in 2009 to encourage development and production of affordable vaccines for developing countries. The overarching objectives of the pneumococcal AMC is to reduce morbidity and mortality from pneumococcal diseases, preventing an estimated 7 million childhood deaths by 2030.^3^ In 2010, the 2 PCV manufacturers agreed to supply PCV10 and PCV13 at reduced pricing for use by low income countries (LIC) through this mechanism. Following assessment of PCV introduction feasibility into the existing vaccine delivery programs, Gavi countries are approved and can access PCVs at the reduced price. The AMC has resulted in incorporation of expanded PCVs into vaccination programs in LIC that were almost simultaneous to introduction in the high income countries, thus avoiding the usual 15–20 y lag in new vaccine introductions in LICs.^4^ Nicaragua was the first Gavi country to participate in the pneumococcal AMC introducing PCV13 in December 2010, the same year of its approval and use in the US and other countries. The WHO renewed its recommendation for PCV introduction in 2012.^5^ Of the 73 countries eligible for Gavi support, 50 have introduced PCV and an additional 8 Gavi countries have been for introduction in the coming years.^6^ By the end of 2015 it is estimated that 80% of GAVI-eligible countries will have introduced PCVs.^3^

Evaluation following PCV introduction is key to understanding PCV impact in developing countries which may differ from those in high income countries. Impact on mortality, invasive pneumococcal disease (IPD), pneumonia and nasopharyngeal carriage (NPC) are important endpoints in the assessment of PCV programs in the developing world. Fifteen Gavi countries have at least one ongoing study evaluating PCV effectiveness (**Table 1**)^3,7^ and currently available data from developing countries are described below.

**Table 1.** PCV impact studies in Gavi countries

Country	Vaccine	Study Endpoints^*^
Bangladesh	PCV10	IPD, Pneumonia, NPC
Burkina Faso	PCV13	Meningitis, Pneumonia, NPC
Gambia	PCV13	IPD, Pneumonia, NPC
Haiti	PCV13	Meningitis, NPC
Kenya	PCV10	IPD, NPC
Lao PDR	PCV13	Pneumonia, NPC
Mongolia	PCV13	IPD, Pneumonia, NPC
Mozambique	PCV10	IPD, Pneumonia, NPC
Malawi	PCV13	Pneumonia, NPC
Nepal	PCV10	IPD, Pneumonia, NPC
Pakistan	PCV10	IPD, NPC
Papua New Guinea	PCV13	IPD, Pneumonia, NPC
Rwanda	PCV13	Meningitis, Pneumonia
Togo	PCV13	IPD, Pneumonia, NPC
United Republic of Tanzania	PCV13	NPC

* IPD-invasive pneumococcal disease, NPC-nasopharyngeal carriage

Evaluation of IPD with serotype-specific data is the gold standard for effectiveness measurement and allows assessment of serotype-specific effectiveness and serotype replacement. Surveillance of IPD is particularly challenging because it involves obtaining sterile site specimens (i.e., blood, cerebrospinal fluid, pleural fluid) which may not be routinely cultured, and if done may be negatively influenced by antimicrobial pretreatment. IPD also represents only a small fraction of PCV preventable PD.^8^ Despite these challenges, evidence demonstrates that PCVs, both PCV10 and PCV13, are effective for prevention of IPD. South Africa, a middle income country with high levels of PD and mortality and high incidence of HIV infection introduced PCV7 in a 2 + 1 dosing schedule (doses at 6 wks, 14wks and 9mo) in 2009 and PCV13 replaced PCV7 in 2011 without catch-up. With approximately 81% compliance with the 3 doses in 2012, a nationwide IPD surveillance network demonstrates effectiveness very similar than that seen in high income countries.^9^ An overall reduction of 69% in IPD was demonstrated in children < 2 y of age; serotype-specific significant decreases in this age group were seen for each of the PCV7 serotypes and for PCV13 serotypes 1, 3, 6A and 19A.^10^ Reductions in PCV7 IPD were similar, 85% and 86% in HIV-uninfected and HIV-infected children < 2 y of age, respectively. Despite the decrease in HIV-infected children, their burden remains 9.8 times higher than that of HIV-uninfected children. Serotype replacement was only significant in the subset of HIV-uninfected children <2 y of age. Significant decreases (>80%) were also seen in penicillin-nonsusceptible, ceftriaxone-nonsusceptible and multidrug-resistant isolates. Importantly, significant reductions in IPD were seen in several unimmunized age cohorts, (infants <10 weeks of age, children 2–4, 5–9 and in adults aged 15–24 and 25–44 years) demonstrating herd protection in these unvaccinated groups including significant reductions in HIV-infected adults aged 25–44 y and reductions in antibiotic nonsusceptible isolates in all age groups. Kenya introduced PCV10 in January 2011 using a 3 + 0 regimen with doses at 6, 10 and 14 weeks of age. Catch-up to 5 y of age was conducted in the Kilifi region which has a robust IPD surveillance system.^11,12^ By the end of 2013, disease due to PCV10 serotypes has decreased approximately 85%. All-serotype IPD in children aged < 5 y had decreased by approximately 70%, but an increase was seen in 2014, with total IPD reduction, thus far, of approximately 30%. The Gambia introduced PCV7 in 2009 and PCV13 replaced it in 2011, using a 3 + 0 regimen at 2, 3, and 4 months without catch-up; compliance is documented to be approximately 90% with the third dose. Thus far, the incidence of PCV13 serotype IPD has decreased from >200 cases /10^5^ to <50 cases/10^5^ in the vaccine target group of 2–23 month old children. (Grant Mackenzie, personal communication) Malawi introduced PCV13 in November 2011 using a 3 + 0 schedule at 6, 10 and 14 weeks; surveillance of meningitis comparing 2 y post-introduction to the pre-introduction years by time-series evaluation model demonstrated lower than predicted numbers of infant meningitis cases, but no reduction on the predicted number of cases in adults was seen after just 2 y of follow-up.^13^

Although pneumonia is the most common cause of pneumococcal disease mortality, surveillance for PCV impact on pneumonia is particularly challenging. Proving the pneumococcal etiology of pneumonia is difficult; there is no easily obtainable diagnostic test and isolation of SP occurs in the minority of pneumococcal pneumonia cases. Therefore, surveillance of presumed bacterial pneumonia and/or all-cause pneumonia is frequently performed and data are available for PCV10 and PCV13 in developing countries. A case-control study in HIV-uninfected children 16–103 weeks of age in Soweto, South Africa demonstrated >50% effectiveness of PCV7 followed by PCV13 on hospitalization for presumed bacterial pneumonia.^14^ Use of administrative databases to compare rates of diseases known to be caused by pneumococcus, pre- and post- PCV introduction, have been used for impact evaluation. When a control diagnosis, known not to be affected by PCV vaccination, is included results may be highly suggestive of PCV impact. Nicaragua used this approach to assess PCV13 impact. PCV13 was introduced in a 3 + 0 schedule at 2, 4 and 6 month of age with a catch-up dose provided to children aged 12–24 months; compliance with the 3 dose schedule was >90%. Two years following PCV13 introduction pneumonia hospitalizations were significantly reduced by 33% and 26% in children < 12 months of age and 12–23 months of age, respectively.^15^ In addition, decreased seasonality of pneumonia cases, was also documented, possibly indicating a decreased incidence of postviral pneumococcal pneumonia. Evaluation of health statistics data on pneumonia in 2 regions of Kazakhstan that introduced PCV13 in a 2 + 1 schedule at 2, 4 and 12–15 months of age, demonstrate reductions of 46% and 49.6% in the target vaccine age group.^16^ In Brazil where the use of administrative databases has been validated for PCV impact assessment,^17^ implementation of PCV10 in a 3 + 1 schedule at 2, 4, 6 and 12 months with catch-up to 24 months of age, demonstrated statistically significant decreases (23.3 to 28.7%) in pneumonia hospitalizations in children 2−24 months of age one year following PCV introduction, in 3 of 5 regions evaluated.^18^ Evaluation, 2 y post PCV10 implementation, demonstrates a 12.65% decrease in pneumonia diagnoses in children 1–4 y of age.^19^

NPC of the pneumococcus is known to be a precursor to invasive and mucosal PD. PCVs have demonstrated ability to reduce carriage of serotypes contained in the vaccine, although overall pneumococcal carriage may be only slightly decreased or unchanged. Decrease in NPC of vaccine-serotypes (VT) results in decreased transmission of these serotypes and consequently, less VT disease, not only in the immunized child but also in unimmunized adults and children. This phenomenon, known as indirect or herd protection has been extensively documented for PCV7 and data regarding herd effect for PCV10 and PCV13 is emerging. Based on this premise and the ease of obtaining specimens, NPC has been used as a surrogate for measuring PCV impact. Reduction in VT NPC in the developing world has been seen for PCV10 in Kenya (3 + 0) and for PCV13 in The Gambia (3 + 0) and South Africa (2 + 1). Although it is speculated that introduction with a catch-up program will accelerate herd effect, recent data in Kenya, demonstrate reduction of VT NPC in unvaccinated children and adults in Kilifi where a PCV10 catch-up program up to 5 y of age was implemented, as well as in unvaccinated children aged 1–4 in Kibera where there was no catch-up program.^12,20^

Although the isolation of a pneumococcus from NPC in a child with pneumonia is not necessarily predictive of a pneumococcal etiology of their pneumonia, the absence of a VT in NPC in a child with pneumonia will likely reflect vaccine effectiveness over time in preventing pneumococcal pneumonia episodes due to that serotype. Thus, the longitudinal monitoring of NPC in children with pneumonia may be a useful and relatively inexpensive way to monitor vaccine impact in children. The collection of data on vaccine exposure in these children with pneumonia may also allow an assessment of the onset of herd protection when the incidence of VT carriage in vaccine-exposed and vaccine-naïve infants would be expected to approximate each other. The principle of using NPC in infants to document onset of herd protection has been established in Massachusetts, albeit in children without a specific pneumonia diagnosis.^21^ In addition, collection of NPC from children with pneumonia may increase the likelihood of detection of more invasive serotypes, such as types 1 and 5 in carriage.^22^

Early data from developing world countries demonstrate a high degree of effectiveness, comparable to that seen in high income countries and should encourage countries who have yet to incorporate PCVs into their national immunization programs to do so. Given the high pneumococcal disease burden and mortality in the developing world, these data translate to enormous public health benefit. As evidence for herd protection accumulates in both the developing and developed world, it may be possible to modify existing PCV immunization programs to maintain herd protection, with less emphasis on the need for individual protection of infants, once the circulation of vaccine types globally has been greatly reduced.

## Disclosure of Potential Conflicts of Interest

No potential conflicts of interest were disclosed.
